# Involvement of MicroRNAs in the Regulation of Muscle Wasting during Catabolic Conditions*

**DOI:** 10.1074/jbc.M114.561845

**Published:** 2014-06-02

**Authors:** Ricardo José Soares, Stefano Cagnin, Francesco Chemello, Matteo Silvestrin, Antonio Musaro, Cristiano De Pitta, Gerolamo Lanfranchi, Marco Sandri

**Affiliations:** From the ‡ Dulbecco Telethon Institute, Venetian Institute of Molecular Medicine, 35129 Padova, Italy,; the Department of § Biomedical Sciences and; the Department of ‖ Biology and CRIBI Biotechnology Centre, University of Padova, 35121 Padova, Italy,; the ¶ Ph.D. Programme in Experimental Biology and Biomedicine, Center for Neuroscience and Cell Biology, University of Coimbra, 3004-517 Coimbra, Portugal,; the ‡‡ Institute of Neuroscience, Consiglio Nazionale delle Ricerche, 35121 Padova, Italy,; the ** DAHFMO-Unit of Histology and Medical Embryology, Sapienza University, 00161 Roma, Italy, and; the §§ Telethon Institute of Genetics and Medicine (TIGEM), 80131 Napoli, Italy

**Keywords:** Gene Expression, MicroRNA (miRNA), Muscle, Muscle Atrophy, Pathology, Protein Degradation, Signal Transduction, Skeletal Muscle, Transcription

## Abstract

Loss of muscle proteins and the consequent weakness has important clinical consequences in diseases such as cancer, diabetes, chronic heart failure, and in aging. In fact, excessive proteolysis causes cachexia, accelerates disease progression, and worsens life expectancy. Muscle atrophy involves a common pattern of transcriptional changes in a small subset of genes named atrophy-related genes or atrogenes. Whether microRNAs play a role in the atrophy program and muscle loss is debated. To understand the involvement of miRNAs in atrophy we performed miRNA expression profiling of mouse muscles under wasting conditions such as fasting, denervation, diabetes, and cancer cachexia. We found that the miRNA signature is peculiar of each catabolic condition. We then focused on denervation and we revealed that changes in transcripts and microRNAs expression did not occur simultaneously but were shifted. Indeed, whereas transcriptional control of the atrophy-related genes peaks at 3 days, changes of miRNA expression maximized at 7 days after denervation. Among the different miRNAs, microRNA-206 and -21 were the most induced in denervated muscles. We characterized their pattern of expression and defined their role in muscle homeostasis. Indeed, *in vivo* gain and loss of function experiments revealed that miRNA-206 and miRNA-21 were sufficient and required for atrophy program. *In silico* and *in vivo* approaches identified transcription factor YY1 and the translational initiator factor eIF4E3 as downstream targets of these miRNAs. Thus miRNAs are important for fine-tuning the atrophy program and their modulation can be a novel potential therapeutic approach to counteract muscle loss and weakness in catabolic conditions.

## Teaser

**Background:** MicroRNAs are important modulators of gene expression but their role in the atrophy program and in muscle loss is unknown.

**Results:** miRNA-206 and miRNA-21 are critical for regulation of the atrophy program after denervation.

**Conclusion:** miRNAs are important for the fine-tuning of the atrophy program.

**Significance:** Modulating miRNA expression is a novel potential therapeutic approach for counteracting muscle loss and weakness in catabolic conditions.

## Introduction

Skeletal muscle is a major site of metabolic activity and the most abundant tissue in the human body accounting for almost 50% of the total body mass. Being the largest protein reservoir, muscle serves as a source of amino acids to be utilized for energy production by various organs during catabolic periods (1). For instance, amino acids generated from muscle protein breakdown are utilized by the liver to produce glucose and support acute phase protein synthesis (1). A number of catabolic disease states, including sepsis, burn injury, cancer, AIDS, diabetes, heart and renal failure are characterized by muscle wasting, mainly reflecting increased breakdown of myofibrillar proteins. Loss of muscle proteins results in muscle atrophy and weakness that have significant clinical consequences resulting in bad prognosis and life expectancy. Conversely, some forms of physical activity such as endurance exercise prevent a decrease of skeletal muscle mass and induces clinical benefits for patients (2–4). However, the molecular mechanisms and the signaling pathways that control muscle strength and size are largely unknown and only recently have some of them been unraveled.

Skeletal muscle mass is sustained by a balance between accumulation of newly synthesized proteins and degradation of existing proteins. Different pathways regulate the balance of these two processes and affect muscle performance (5). This view was recently advanced by the finding that muscle atrophy requires a transcriptional regulation of a subset of key genes. The analysis of the gene expression profiles of muscle collected from different atrophic models (*i.e.* diabetes, cancer cachexia, chronic renal failure, fasting, and denervation) led to the identification of a subset of genes that are commonly up- or down-regulated in muscle atrophy. Because all these diseases share muscle atrophy as a common trait, these genes were called atrophy-related genes or atrogenes and are believed to regulate the loss of muscle components (6). These findings indicate that muscle atrophy is an active process controlled by specific signaling pathways and transcriptional programs. In fact, several transcription factors have been found to regulate muscle atrophy (7–12). Considering the complexity of the atrophic program, other levels of regulation may be involved.

MicroRNAs (miRNAs)^5^ are endogenous ∼22 nucleotide small noncoding RNAs that control gene expression by targeting mRNAs and triggering either translation repression or RNA degradation. These noncoding RNAs bind to the 3′UTR of the target mRNA, and the specificity of the binding is dictated mainly by the seed region of the miRNA (from nucleotide 2 to 8). Hundreds of target genes, often belonging to the same pathway, are regulated by the same miRNA. To increase the complexity of this post-transcriptional regulation several miRNAs can target the same transcript (13).

miRNAs are required for several biological processes and complete lack of miRNAs is incompatible with life (14, 15). Interestingly, many miRNAs are expressed in a tissue-specific manner (16). This is the case of several miRNA specifically expressed in cardiac and skeletal muscles and, therefore, named myo-miRs (16–18). In striated muscles, miRNAs are involved in physiological processes such as myogenesis, fiber-type switch, and regeneration (19–21). Consequently, deregulation of the miRNA expression levels is associated with pathological conditions (22, 23). For instance, inhibition of miRNA-206 in SOD1^G93A^ transgenic mice induced severe atrophy (24). However, the involvement of miRNAs in skeletal muscle atrophy is largely unknown.

To determine which miRNAs are relevant for the atrophic process, we performed miRNA expression profiling in muscles from different atrophic models including fasting, denervation, streptozotocin-induced diabetes, and cancer cachexia. We checked whether there was a common signature of miRNA expression in different atrophying conditions and found that each catabolic situation is associated to a peculiar set of deregulated miRNAs. We further focused on atrophy induced by denervation and identified the two most up-regulated miRNAs, miRNA-206 and miRNA-21. *In vivo* loss- and gain-of-function experiments defined the functional relevance of these two miRNAs for muscle mass regulation. Finally, by combining gene expression profiling and bioinformatic prediction tools we identified and characterized two downstream targets of these atrophy miRNAs.

All together, these results start to unravel, *in vivo*, the role of miRNAs in the atrophic process and open the possibility to manipulate microRNA expression as a novel therapeutic approach to counteract muscle loss in catabolic conditions.

## EXPERIMENTAL PROCEDURES

### 

#### 

##### Animal Models and Surgical Procedures

All experimental procedures were approved and authorized by the Italian Ministry of Health. Mice were housed in individual cages in an environmentally controlled room (23 °C, 12-h light-dark cycle) and provided food and water *ad libitum*.

Four different models of skeletal muscle wasting were used: starvation, denervation, diabetes, and cancer cachexia. Starvation, denervation, and diabetes were performed on adult, 2-month-old, CD1 mice, whereas cancer cachexia was induced on 7-week-old BALB/c mice.

Fasting was performed as previously described, by removing chow with free access to water. Denervation experiments were performed by cutting the sciatic nerve of one limb, whereas the other was used as control. Mice were sacrificed after 3, 7, or 14 days after denervation. Diabetes was induced by one single acute intraperitoneal (IP) injection of 180 mg/kg of streptozotocin (Sigma S-0130). To induce cancer-associated cachexia, a 0.5-mm^3^ solid fragment of colon carcinoma (C26, obtained from the National Cancer Institute) was subcutaneously implanted in the back of 7-week-old BALB/c mice (Charles River). Mice were killed 14 days after tumor injection, when the tumor bearing mice had a body weight loss of ∼25% compared with control mice, and hindlimb muscles were removed. For this model mice were housed individually in the Animal Care Facility at the Unit of Histology and Medical Embryology (Rome).

##### After Animal Death Muscles Were Collected and Immediately Frozen in Liquid Nitrogen for Further Analysis

Tibialis anterior (TA) muscles of adult CD1 male mice (28–34 g) were transfected as described previously (9, 25). Briefly, plasmid DNA was injected along the muscle length. Electric pulses were then applied by two stainless steel spatula electrodes with the Electro Square Porator (ECM 830, BTX). Transfected muscles were collected 7, 10, or 14 days after electroporation.

##### Cell Culture

For *in vitro* experiments we used C2C12 (mouse myoblast) cells, bought from ATCC. Cells were maintained in culture with DMEM (Dulbecco's modified Eagle's medium) containing 10% FBS and 1% penicillin-streptomycin at 37 °C and 5% CO_2_. C2C12 cells were maintained in proliferation and were not differentiated into myotubes. Transfection was performed using the Lipofectamine 2000 reagent (Invitrogen 11668-027) according to the manufacturer's protocol. One day prior to the transfection, cells were plated onto 6-well plates at a density of 100,000 cells per well. Cells were transfected with a total of 4 μg of plasmidic DNA per well (6-well plate). Cells were used 24 or 48 h after transfection, according to the experiments.

##### RNA and miRNAs Purification

For the microarray experiments mRNA and miRNAs were isolated from frozen gastrocnemius muscles. miRNAs/mRNAs isolation was achieved by an initial purification with TRIzol (Invitrogen) followed by subsequent purification and fractionation with the Purelink miRNA Isolation Kit (Invitrogen number K1570-01). Total and small RNA were quantified using the ND-1000 spectrophotometer (Nanodrop), whereas RNA integrity and content of microRNAs (%) in each sample were assessed by capillary electrophoresis using the RNA 6000 Nano LabChip and the Small RNA Nano LabChip, respectively, using the Agilent Bioanalyzer 2100 (Agilent Technologies). Only total RNA samples with RNA integrity number values >6, and the percentage of miRNA <20% were used for microarray analysis.

##### miRNAs Expression Profiling

The miRNA expression profiles were generated using our in-house printed array containing the mirVana miRNA Probe Set (Ambion; catalog number 1564V1). Negative control probes were designed using sequences from *Escherichia coli*; positive control probes (spikes) are sequences from *Mytilus galloprovincialis* (GEO platform number GPL17835).

Small RNA molecules (<200 nucleotides) were labeled with the mirVana labeling kit and amine-reactive dyes as recommended by the manufacturer (Ambion). Briefly, poly(A) polymerase and a mixture of unmodified and amine-modified nucleotides were used first to append a polynucleotide tail to the 3′-end of each miRNA. The amine-modified miRNAs were then cleaned up and coupled to NHS ester-modified Cy5 or Cy3 dyes (GE Healthcare). Unincorporated dyes were removed with a second glass fiber-based clean-up procedure as described (26).

Microarray hybridization was carried out in a dual slide chamber (HybChamber, Gene Machines) humidified with 100 μl of 3× SSC. Labeled RNA was dissolved in 6 μl of 3× miRNA Hybridization Buffer (Ambion), denatured at 95 °C for 3 min, and applied directly to the slides. Microarrays were covered with a 24 × 24-mm coverslip and hybridized for 21 h at 42 °C by immersion in a high precision water bath (W28, Grant). Hybridized slides were successively washed in low and high stringency buffer (Ambion) for 30 s and dried by centrifugation (500 × *g*). Array scanning was carried out using a GSI Lumonics LITE dual confocal laser scanner with a ScanArray Microarray Analysis System (PerkinElmer Life Sciences), and raw images were analyzed with QuantArray Analysis Software (GSI Lumonics).

##### Gene Expression Profiling

The Mouse Genome Oligo Set (version 1.1, Operon) consisted of 13,439 70-mer oligonucleotide probes purchased from the Microcribi-Gene Expression Service available at CRIBI (University of Padova). Each oligonucleotide was spotted in two replicates on MICROMAX SuperChip I glass slides (Perkin-Elmer) using Biorobotics Microgrid II (V&P Scientific, Inc.). We produced an updated and careful annotation of all sequences by querying three databases: ENSEMBL (version 56), RefSeq (version 38), and UniGene (version 183). About 1,500 probes did not find significant hits. The updated platform (version 2.0) has been submitted to the GEO Database with accession number GPL10688.

Total RNA (>200 nucleotides) samples were labeled using the Amino Allyl MessageAmp^TM^ II aRNA Amplification Kit (Ambion, Austin, TX), in accordance with the manufacturer's instructions. Briefly, first strand synthesis with an engineered reverse transcriptase should produce virtually full-length cDNA, which is the best way to ensure reproducible microarray results. The use of a modified oligo(dT) primer bearing a T7 promoter (27) allows the next amplification steps: after second strand synthesis and clean-up the cDNA becomes a template for *in vitro* transcription with T7 RNA polymerase. About 5 μg of aminoallyl-labeled aRNA were coupled with Cy5 or Cy3 dyes (GE Healthcare) and purified on a column (Ambion). Labeled targets (atrophic and control muscle) were mixed and ethanol precipitated. After dissolving the pellet in 120 μl of hybridization buffer (5× SSC, 0.1% SDS, 25% formamide), samples were denatured at 90 °C for 2 min and added to the microarrays. Prehybridization was performed overnight at 48 °C in the presence of 5× SSC, 5× Denhardt, 0.1% SDS, 100 ng/μl of single-stranded DNA. Competitive hybridizations were carried on for 48 h at 46 °C in an ArrayBooster microarray incubator (Advalytix), followed by a series of post-hybridization washings (28). Slides were scanned on a GSI Lumonics LITE dual confocal laser scanner (PerkinElmer Life Sciences) and QuantArray software (GSI Lumonics) was used for image analysis. Raw data are available on GEO database using accession number GSE52676.

##### Statistical Analysis of miRNA and Gene Expression Data

Interarray normalization of expression levels performed with loessM+GPA for miRNA experiments and a within-array-normalization, using the lowess method, was performed by using the MIDAW tool for gene expression profiling (29). Normalization function was applied to miRNA expression data of all experiments and then values of spot replicates within arrays were averaged.

Cluster analysis and profile similarity searching were performed with tMev that is part of the TM4 Microarray Software Suite. In particular, hierarchical cluster analysis was performed with a Euclidean distance coefficient as distance measure with complete linkage. One-way analysis of variance was used to identify miRNAs differentially expressed (*p* value ≤ 0.05 based on 1,000 permutation). Pathway analysis was performed using the WEB-based GEne SeT AnaLysis Toolkit (30) and KEGG database on differentially expressed genes of denervated muscles compared with controls through SAM analysis (31). Benjamini and Hochberg multiple test adjustment was used to the determine pathway significance and the top 10 pathways were considered.

##### Identification of miRNA Target Genes

One of the most important and difficult steps in miRNA investigations is the identification of their target genes to understand the mechanism by which miRNAs can modulate a certain biological function. We have first recovered potential mRNA targets calculated by the PITA algorithm (32). Hundreds of target genes for any single miRNA have been predicted by this analysis. To limit the fraction of false positives we have analyzed the functional anticorrelation between miRNAs and mRNA expression levels (*e.g.* miRNA is up-regulated and corresponding mRNA target is down-regulated) (Fig. 5*A*) by searching specific profiles of miRNAs and mRNAs through the template matching algorithm (33). Gene ontology classification of miRNA-206 and -21 targets was performed using the DAVID web tool (34).

##### Validation of the Microarray Results

miRNA expression profiles were validated using the TaqMan® MicroRNA Assays (Applied Biosystems). Retrotranscription of each specific miRNA was accomplished using the TaqMan MicroRNA Reverse Transcription Kit (Applied Biosystems) according to the manufacturer's instructions. RT-PCR was then preformed using the TaqMan Universal PCR Master Mix and the specific primers from TaqMan MicroRNA Assay specific for each miRNA. miRNAs expression was normalized with the U6 small nuclear RNA (U6 snRNA) (Applied Biosystems, assay ID 001973). Studied miRNA were miRNA-21 (Applied Biosystems, assay ID 000397), miRNA-206 (Applied Biosystems, assay ID 000510), and miRNA-133b (Applied Biosystems, assay ID 002247).

Gene expression analysis was performed by qRT-PCR. Complementary DNA was generated using Superscript III Reverse transcriptase (Invitrogen number 18080044). cDNA was amplified, using Power SYBR Green PCR Master Mix (Applied Biosystems number 4367659), with an ABI Prism 7900HT (Applied Biosystem) thermocycler. Gapdh and Actin were used as a reference to normalize expression data. The sequence of the oligonucleotide primers used is shown in Table 1.

**TABLE 1:** **List of primers used for real time experiments**

Primer	Sequence
GAPDH-forward	5′-CACCATCTTCCAGGAGCGAG-3′
GAPDH-reverse	5′-CCTTCTCCATGGTGGTGAAGAC-3′
Pan-Actin-forward	5′-CTGGCTCCTAGCACCATGAAGAT-3′
Pan-Actin-reverse	5′-GGTGGACAGTGAGGCCAGGAT-3′
Yy1-forward	5′-TGAGAAAGCATCTGCACACC-3′
Yy1-reverse	5′-CGCAAATTGAAGTCCAGTGA-3′
Eif4e3-forward	5′-AACATCCCTCCTGTGACCAG-3′
Eif4e3-reverse	5′-TCCAATGGTCGCTAACAACA-3′
Pdcd10-forward	5′-GGGCACTTGAACACCAAAAG-3′
Pdcd10-reverse	5′-CAGGCCACAGTTTTGAAGGT-3′

##### Cloning and Plasmids

Transfection and electroporation experiments were preformed with the following constructs. pmiRZIP lentivector (ZIP NULL), pmiRZIP lentivector anti-miRNA-206 (ZIP-206), and pmiRZIP lentivector anti-miRNA-21(ZIP-21) were acquired from System Bioscience. pMIR206-Luc and pMIR21-Luc were acquired from Signosis BioSignal. *Renilla* Null and *Renilla* TK were acquired from Promega (pRL-null number E2271, pRL-TK number E2241).

The mature sequences of miRNA-206 and miRNA-21 were cloned into the BLOCK-iT Pol II miR RNAi Expression Vector Kit with EmGFP (Invitrogen number K4936-00). The oligos used are shown in Table 2. As negative control the pcDNA6.2-GW/EmGFP-miR-neg control was used, according to manufacturer's instructions.

**TABLE 2:** **List of primers used in cloning experiments**

Name	Sequence
miRNA-206, top	5′-TGCTGTGGAATGTAAGGAAGTGTGTGGGTTTTGGCCACTGACTGACCCACACACCC TACATTCA-3
miRNA-206, bottom	5′-CCTGTGAATGTAGGGTGTGTGGGTCAGTCAGTGGCCAAAACCCACACACTTCCTTACATTCCAC-3
miRNA-21, top	5′-TGCTGTAGCTTATCAGACTGATGTTGAGTTTTGGCCACTGACTGACTCAACATCTCTGATAAGCTA-3′
miRNA-21, bottom	5′-CCTGTAGCTTATCAGAGATGTTGAGTCAGTCAGTGGCCAAAACTCAACATCAGTCTGATAAGCTAC-3′
Yy1-3′UTR-forward	5′-GCCTCTTCAGGAGTGTGATTG-3′
Yy1-3′UTR-reverse	5′-CAATTTCTGGGAGGCTCAAG-3′
Eif4e3-3′UTR-forward	5′-TCTGCCATCGTATCACTTGC-3′
Eif4e3-3′UTR-reverse	5′-GCCTCTTACGCTCTGACCAC-3′
Pdcd10-3′UTR-forward	5′-ACTAGTCCAGGATGTTGAATGGGATT-3′
Pdcd10-3′UTR-reverse	5′-GCGGCCGCAAGTAAAGAAATGTTTAACA-3′
PolK-3′UTR-forward	5′-ACTAGTCCTTTAAGGAAGACAAGTGCAA-3′
PolK-3′UTR-reverse	5′-GCGGCCGCCAACAAAAATAAACTTCAGATGGAA-3′

The 3′UTR of the different analyzed genes was cloned from muscle cDNA into the pMIR-LUC vector (Signosis BioSignal). The sequence of the oligonucleotide primers used to clone the 3′UTR of each gene are shown in Table 2. All constructs were sequenced to confirm the insert and absence of mutation.

To mutate the 3′UTR of YY1 and eIF4E3 the QuikChangeII site-directed Mutagenesis Kit (Stratagene number 200524) was used according to the manufacturer's instructions. The primers used for the mutagenesis are shown in Table 3. All mutated constructs were sequenced to confirm the presence of the desired mutations and the absence of other unspecific mutations.

**TABLE 3:** **List of primers used for the mutagenesis experiments**

Primer	Sequence
Eif4e3_mut-206_forw	5′-AAAGTCAGGGGCCTCCACTTGAAGCGCTAAACAGGAAGCCAAATTA-3′
Eif4e3_mut-206_rev	5′-TAATTTGGCTTCCTGTTTAGCGCTTCAAGTGGAGGCCCCTGACTTT-3′
Eif4e3_mut-21_forw	5′-GCCTAGCAAAACCCTTTTTCTGCTGCATTGTTGTGACACTTCCCTGCA-3′
Eif4e3_mut-21_rev	5′-TGCAGGGAAGTGTCACAACAATGCAGCAGAAAAAGGGTTTTGCTAGGC-3′
Yy1_mut-1_forw	5′-GTGCATATTGTACACTTTTTGGGGATCTTATTAGTAATGCTGTGTGATTTTCTGGA-3′
Yy1_mut-1_rev	5′-TCCAGAAAATCACACAGCATTACTAATAAGATCCCCAAAAAGTGTACAATATGCAC-3′
Yy1_mut-2_forw	5′-GCTGTGTGATTTTCTGGAGGTTGATCGCTGTGCTTGCGGTAGATTTTCTTT-3′
Yy1_mut-2_rev	5′-AAAGAAAATCTACCGCAAGCACAGCGATCAACCTCCAGAAAATCACACAGC-3′

##### Cross-sectional Area Measurements

Cross-sectional area of GFP-positive transfected fibers was measured as described previously (8) and compared with the surrounding nontransfected myofibers (control). The pictures of the transfected muscles were taken at ×20 magnification with a fluorescence microscope (Zeiss AxioImager.Z1). Fiber cross-sectional areas were measured using IMAGE software (Scion). All data are expressed as the mean ± S.E. Comparison were made by using the Student's *t* test, with *p* < 0.05 being considered statistically significant.

##### Luciferase Assays

Luciferase measurements in muscles transfected with reporter constructs were performed using the Dual Luciferase Reporter Assay System (Promega number E1910) adapting the manufacturer's instructions. Briefly, muscles were powdered in liquid nitrogen before the addition of the lysis buffer. The suspension was repeatedly frozen and thawed and then centrifuged. The supernatant was analyzed according to the manufacturer's instructions. To control for transfection efficiency, firefly luciferase activity was divided by *Renilla* luciferase activity. Results are expressed as mean ± S.E. of at least three different animals. In cells, luciferase assay was performed according to the manufacturer's instructions.

##### Protein Extraction and Western Blot

C2C12 cells were lysed in 50 mm Tris, pH 7.5, 150 mm NaCl, 5 mm MgCl_2_, 10% glycerol, 1 mm DTT, 1 mm EDTA, 0.5% Triton X-100, Phosphatase inhibitor mixture 2 (Sigma number P5726), and phosphatase inhibitor mixture 3 (Sigma number P0044) and Complete EDTA-free protease inhibitor mixture (Roche number 11836145001). Protein concentration was quantified using the Bradford method (Bio-Rad Protein Assay, number 500-0006).

Muscles were lysed in: 50 mm Tris, pH 7.5, 150 mm NaCl, 5 mm MgCl_2_, 10% glycerol, 2% SDS, 1% Triton X-100, 1 mm DTT, phosphatase inhibitor mixture 2 and phosphatase inhibitor mixture 3, and Complete EDTA-free protease inhibitor mixture. Protein concentration was quantified using the BCA method (BCA Protein Assay Kit, Thermo Fisher Scientific number 23227).

Equal volumes of cell or muscle lysates were loaded and separated on 4–7.5% precast gels (Bio-Rad number 456-1023). Proteins were transferred to Hybond-ECL Nitrocululose membrane (GE Healthcare number RPN303D). Immunoreaction was performed according to the antibody requirements. The following antibodies were used: anti-YY1 (NBP1-46218) from Novus Biologicals and anti-Ago2 (Clone 2D4) from Wako Chemicals Gmbh.

##### Accession Numbers

The gene expression profiling data discussed in this publication have been deposited in the NCBI Gene Expression Omnibus and are accessible through GEO Series accession number GSE51648 for miRNAs and GSE52676 for mRNAs.

## RESULTS

### 

#### 

##### Different Atrophic Conditions Are Characterized by a Specific miRNA Signature

**FIGURE 1.**
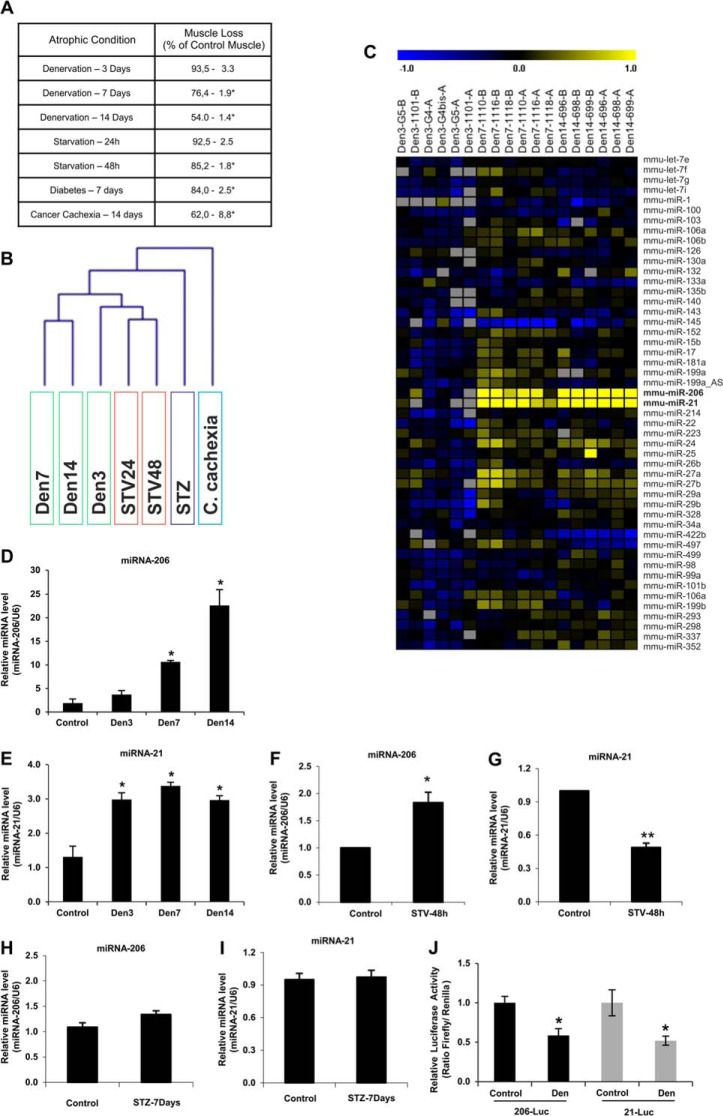
**miRNA expression is deregulated in different models of skeletal muscle atrophy, miRNA-21 and miRNA-206 being the two most induced miRNAs in denervation.** *A,* weight of gastrocnemius muscle in each atrophic condition studied. Data are shown as mean ± S.E.; *n* > 3 muscles per condition. *, *p* < 0.05. *B*, cluster analysis of atrophy conditions. Euclidean distance was used in the clustering. Denervated mice cluster together, separately from starvation, diabetes, and cancer cachexia conditions. *Den3*, denervation 3 days; *Den7,* denervation 7 days; *Den14*, denervation 14 days; *STV24*, starvation 24 h; *STV48*, starvation 48 h; *STZ*, diabetes induced by streptozotocin (*STZ*). *C,* heat map of miRNA differentially expressed between the different times of denervation. The indicated miRNAs functionally characterized are in *bold*. Color code is indicated as log2 (atrophy/control). *Gray box* represents data not available because under limit of detection. *D*, miRNA-206 expression levels are increased at different time points of denervation. qRT-PCR of miRNA expression levels in gastrocnemius of control and denervated animals at different time points. Data are shown as mean ± S.E.; *n* > 3 muscles per condition. *, *p* < 0.05. *E,* miRNA-21 expression levels are increased at different time points of denervation. qRT-PCR of miRNA expression levels in gastrocnemius of control and denervated animals at different time points. Data are shown as mean ± S.E.; *n* > 3 muscles per condition. *, *p* < 0,05. *F,* 48 h of fasting increases the miRNA-206 expression level. qRT-PCR of miRNA expression levels in gastrocnemius of fed and starved animals for 48 h. Data are shown as mean ± S.E.; *n* > 3 muscles per condition. *, *p* < 0.05. *G,* 48 h of fasting decreases the miRNA-21 expression level. qRT-PCR of miRNA expression levels in gastrocnemius of fed and starved animals for 48 h. Data are shown as mean ± S.E.; *n* > 3 muscles per condition. **, *p* < 0.01. *H* and *I*, diabetes does not alter the expression levels of miRNA-206 and miRNA-21. qRT-PCR of miRNA expression levels in gastrocnemius of vehicle or streptozotocin-injected animals (7 days after injection). Data are shown as mean ± S.E.; *n* > 3 muscles per condition. *J,* miRNA-206 and -21 luciferase sensors were inhibited in denervated muscles. Data are shown as mean ± S.E. *n* > 4 muscles per condition; *, *p* < 0.05.

Gene expression profiling of atrophic muscles have identified a group of transcripts that were commonly up- or down-regulated in different catabolic conditions. These genes were named atrophy-related genes. To understand if these catabolic conditions also alter miRNA expression we defined the miRNA signatures of different atrophic conditions. The measurements of muscle mass confirmed that different models of atrophy triggered a significant muscle loss (Fig. 1*A*). Competitive microarray hybridization experiments were performed between a pool of miRNAs obtained from control gastrocnemius muscles and miRNAs obtained from gastrocnemius muscles of starved, denervated, diabetic, and cancer bearing mice, respectively. A platform that contains ∼400 probes for known miRNA (miRBase Release 9) was used for these experiments. Importantly, fasting and denervation were analyzed at different time points to understand the kinetic of miRNA response in these atrophic conditions. The comparison of miRNA profiles did not reveal any common changes but instead identified a peculiar miRNA signature for each catabolic condition (Fig. 1*B*). According to the hierarchical clustering dendrogram (Fig. 1*B*) the two different fasting time points were grouped together. Also denervation experiments were grouped together, mainly at 7 and 14 days. Short-term denervation (3 days) presented a peculiar expression profile. At this early time point most miRNAs did not change or were down-regulated when compared with the controls. A completely independent profile was obtained from diabetic muscles (7 days after treatment with STZ) in which the vast majority of the studied miRNAs appeared up-regulated (Table 4). Conversely, most of miRNAs were inhibited in cachectic muscles (Table 4). Interestingly, the findings obtained from denervated muscles indicate that the miRNA regulation is delayed when compared with mRNAs. In fact, whereas after denervation most of the mRNA expression changes peaked at 3 day of denervation (6, 35), the modifications of miRNA expression were maximal at day 7 after denervation (Fig. 1*C*). Among the up-regulated microRNAs, miRNA-21 and miRNA-206 were strongly induced. Fasted and diabetic muscles also showed an induction of miRNA-206 and miRNA-21 expression (Table 4). Therefore, based on these findings we focused our attention on these two microRNAs.

**TABLE 4:**
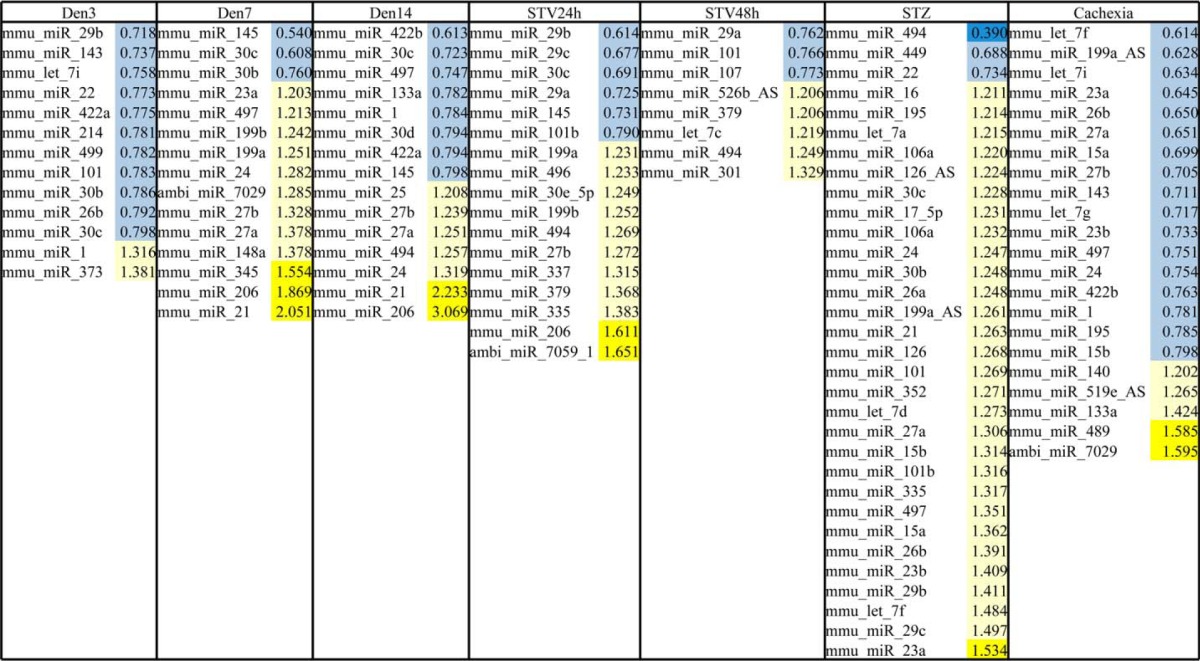
**Differentially expressed miRNAs detected according ANOVA analysis. miRNAs with fold change <0.8 and >1.2 are represented for each atrophic condition (blue is for down-regulation and yellow is for up-regulation)**

Quantitative RT-PCR confirmed the up-regulation of miRNA-206 and miRNA-21 after denervation even if their pattern of expression was slightly different (Fig. 1, *D* and *E*). MicroRNA-206 was also induced by fasting but not by diabetes (Fig. 1, *F* and *H*), whereas miRNA-21 was suppressed by fasting and unchanged in diabetes (Fig. 1, *G* and *I*). Due to the important induction of both of these microRNAs in denervated muscles we focused our attention on this model of muscle atrophy. To further validate miRNA-206 and -21 up-regulation after denervation we used a luciferase-based assay to monitor the activity of the endogenous miRNAs. We used a vector that contains a binding site specific for each miRNA in the 3′UTR of the luciferase gene. When the miRNA of interest is induced, it binds the luciferase transcript blocking its translation. The miRNA-Luc sensors were transfected into TA muscles of adult mice and then animals were denervated. Luciferase activity of miRNA-206 and miRNA-21 sensors was significantly reduced after 7 days of denervation (Fig. 1*J*) confirming the increased activity of these miRNAs after denervation.

**FIGURE 2.**
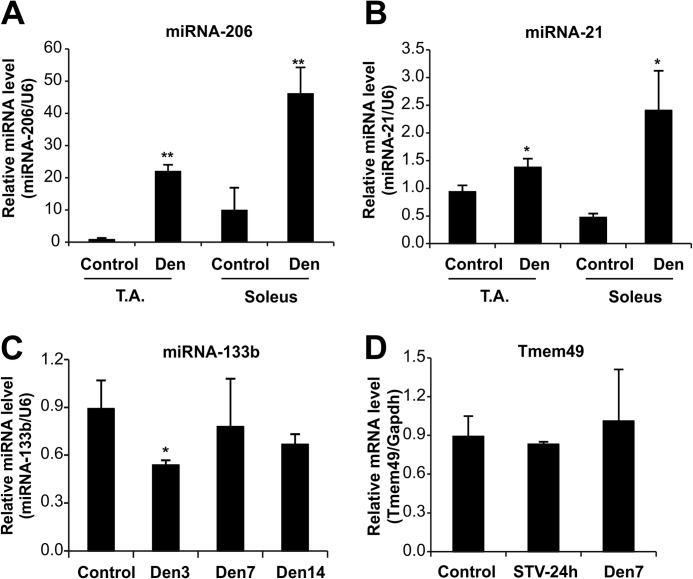
**miRNA-206 and -21 are selectively induced in atrophying muscles.** *A,* miRNA-206, and *B,* miRNA-21 expression levels are increased after 15 days of denervation in both fast and slow muscles. Quantitative RT-PCR analysis on miRNA expression levels in TA and soleus muscles of control and denervated animals. Data are shown as mean ± S.E. *n* > 3 independent experiments; *, *p* < 0.05; **, *p* < 0.01. *C,* expression of miRNA-133b, the co-cistronic partner of miRNA-206, is not up-regulated during denervation. Quantitative RT-PCR analysis on miRNA expression levels in gastrocnemius of control and denervated animals at different time points. Data are shown as mean ± S.E., *n* = 3 independent experiments; *, *p* < 0.05. *D,* expression level of *Tmem49*, the host gene of miRNA-21, during 24 h fasting and 7 days of denervation. Quantitative RT-PCR analysis on mRNA expression levels in gastrocnemius muscles. Data are shown as mean ± S.E., *n* = 3 independent experiments.

Because skeletal muscles contain different fiber types that show different metabolic properties, we monitored miRNA expression in denervated fast and slow muscles. miRNA-206 and miRNA-21 were induced in both TA, a glycolytic mitochondrial poor muscle, and soleus, an oxidative mitochondrial rich muscle (Fig. 2, *A* and *B*) suggesting that miRNA response is a common feature of atrophying muscles.

miRNA-206 is an intergenic polycistronic miRNA embedded in the same genomic region of miRNA-133b, another muscle-specific miRNA (36, 37). Therefore we checked whether miRNA-133b expression was also affected in response to denervation. Interestingly, miRNA-133b was significantly down-regulated at 3 days of denervation, returning to basal level at 7 and 14 days (Fig. 2*C*). These results suggest that miRNA-206 is regulated independently of its polycistronic companion, miRNA-133b.

On the other hand, miRNA-21 is located on the 10th intron of the vacuole membrane protein 1 (VMP1/TMEM49) gene (38). Also in this case the mRNA level of VMP1 remained unchanged in the atrophic conditions indicating that miRNA-21 is transcribed independently from its host gene (Fig. 2*D*).

##### Induction of miRNA-206 and miRNA-21 Contributes to Muscle Atrophy

**FIGURE 3.**
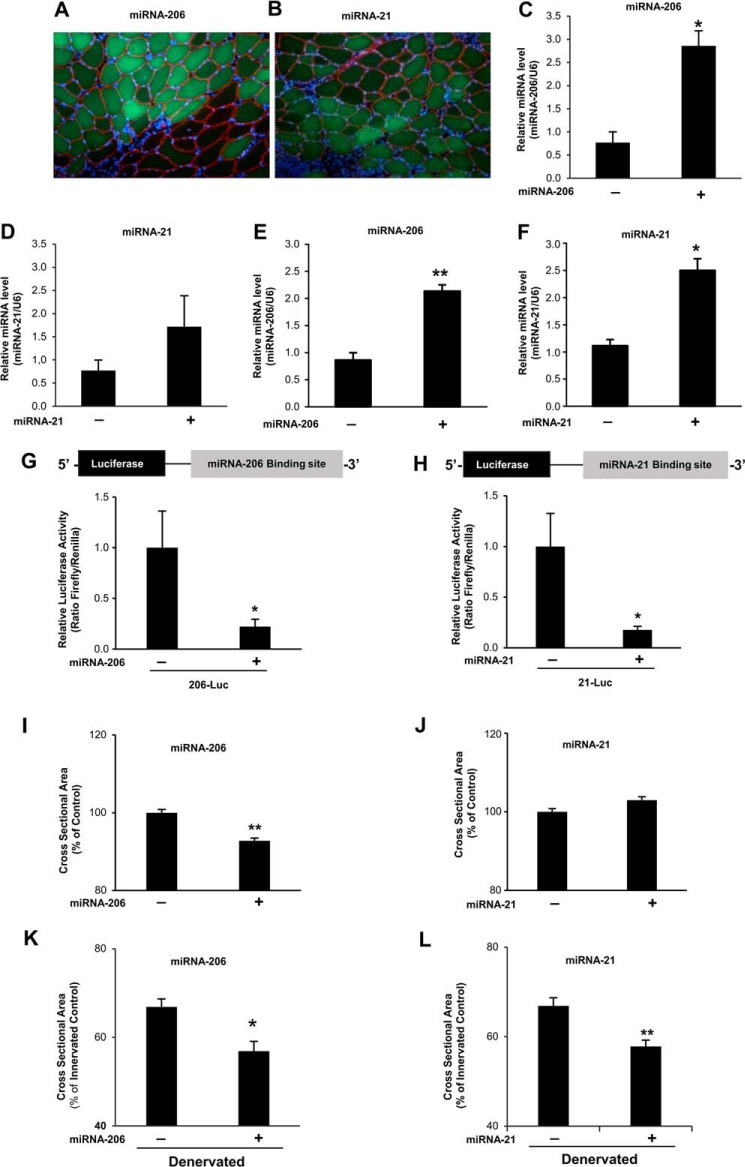
**Over-expression of miRNA-206 and miRNA-21 *in vivo* alters adult muscle fiber size.** *A* and *B, in vivo* transfection of co-cistronic vectors expressing (*A*) miRNA-206 or (*B*) miRNA-21 together and GFP. Adult TA were transfected and collected 10 days after transfection. Cryosections were stained with anti-dystrophin, to identify the plasma membrane of the myofibers, and counterstained with Hoechst. Images were merged to demonstrate that the GFP signal lay under the sarcolemma and not in interstitial cells. *C* and *D*, qRT-PCR of miRNA-206 and miRNA-21 expression levels on transfected TA muscles, respectively. Data are shown as mean ± S.E., *n* > 3 muscles per condition, *, *p* < 0.05. *E* and *F,* qRT-PCR of miRNA-206 and miRNA-21 expression levels on C2C12 myoblasts transfected for 48 h with miRNA overexpressing vectors. Data are shown as mean ± S.E., *n* = 3 independent experiments; *, *p* < 0.05; **, *p* < 0.01. *G* and *H, in vivo* transfection of miRNA-206 or miRNA-21 expressing vectors produces functional mature miRNAs. Adult tibialis anterior muscles were co-transfected with miRNA-expressing vector and with the miRNA luciferase sensor for each miRNA. A *Renilla* luciferase vector was co-transfected to normalize for transfection efficiency. 7 days after transfection, muscles were collected and the luciferase/*Renilla* ratio was determined. Data are shown as mean ± S.E. *n* > 3 muscles per condition; *, *p* < 0.05. *I, in vivo* transfection of miRNA-206 induces skeletal muscle atrophy. Adult TA muscles were transfected and collected 10 days after transfection. Cross-sectional area of transfected fibers, identified by the GFP, was measured. Data are shown as mean ± S.E., *n* > 800 per each muscle, at least 3 muscles per condition were examined; **, *p* < 0.01. *J, in vivo* transfection of miRNA-21 does not affect skeletal muscle fiber size. Transfection and analyses were performed as described in *I. K,* over-expression of miRNA-206 in denervated muscles aggravates the atrophic phenotype. Adult tibialis anterior muscles were transfected with DNA vector over-expressing the mature miRNA-206 and simultaneously denervated. Muscles were collected 10 days after transfection. Analyses were performed as described in *I*, **, *p* < 0.01. *L,* over-expression of miRNA-21 in denervated muscles aggravates the atrophic phenotype. Adult tibialis anterior muscles were transfected with DNA vector over-expressing the mature miRNA-21. Muscles were collected 10 days after transfection. Analyses were performed as described in *I*; *, *p* < 0.05.

To address the biological role of miRNA-206 and miRNA-21, *in vivo*, in adult skeletal muscle we cloned the mature sequence of these miRNAs into a co-cistronic vector that co-express both the miRNA and mRNA for GFP. Adult TA muscles were transfected by electroporation and examined after 8 days. The transfected fibers that express the mature miRNA were easily recognized by GFP fluorescence. The staining for dystrophin revealed that transfection happened only in adult fibers and not in interstitial or satellite cells, the muscle stem cells. Indeed, GFP fluorescence was detected only in dystrophin-positive fibers (Fig. 3, *A* and *B*). Quantitative RT-PCR confirmed that mature miRNA-206 and miRNA-21 were expressed both *in vitro* (Fig. 3, *E* and *F*) and *in vivo* (Fig. 3, *C* and *D*). Importantly, we found a 2–3-fold up-regulation, *in vivo* (Fig. 3, *E* and *F*), suggesting that our transfection technique does not induce massive microRNA production but keeps the expression of mature microRNA close to the pathophysiological conditions, such as denervation.

To further prove that over-expressed miRNA were also functional and able to suppress translation of the target transcript, *in vivo*, we used the luciferase miRNA sensor. Muscles were transfected with the sensor of each miRNA in the presence or absence of the miRNA expression vector. Over-expression of miRNA-206 or miRNA-21 dramatically reduced the luciferase activity confirming that these vectors were able to produce functional miRNAs (Fig. 3, *G* and *H*).

To discriminate whether up-regulation of these miRNAs was sufficient to induce atrophy, we over-expressed miRNA-206 and miRNA-21 either in normal or denervated adult muscle. The expression of miRNA-206 was sufficient to induce a 10% decrease of fiber size in innervated muscles when compared with controls (Fig. 3*I*). Importantly, miRNA-206 over-expression caused a further decrease of fiber size after denervation (Fig. 3*K*). In fact denervated muscle fibers over-expressing miRNA-206 were 10% smaller than denervated scramble-transfected fibers. Therefore, when we anticipate the miRNA-206 induction at the first days after denervation by electroporation, atrophy is exacerbated. These findings suggest that miRNA-206 does not interfere with the atrophy program but instead is part of this process. When miRNA-21 was expressed in innervated muscles, no major differences were observed when compared with controls (Fig. 3*J*). However, after denervation, overexpression of miRNA-21 enhanced muscle atrophy by 11% (Fig. 3*L*).

##### Inhibition of miRNA-206 and miRNA-21 Partially Protects from Denervation-induced Atrophy

**FIGURE 4.**
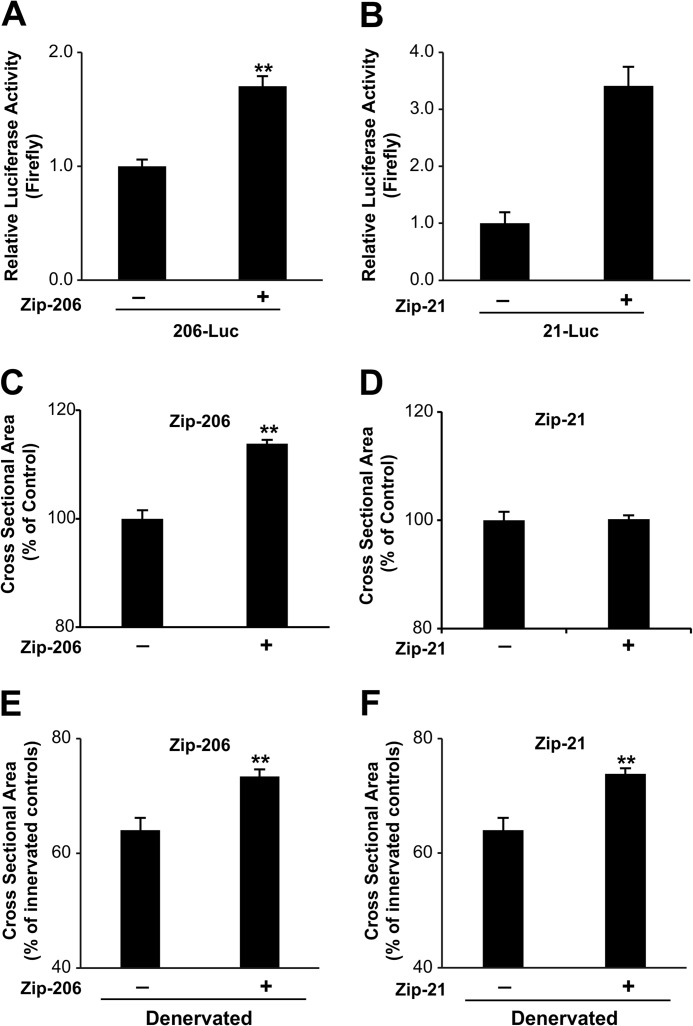
**Inhibition of miRNA-206 *in vivo* induces skeletal muscle hypertrophy.** *A* and *B*, transfection of C2C12 cells with constructs expressing miRNA sponges efficiently inhibits miRNA-206 or miRNA-21. C2C12 myoblasts were transfected with miRZIP and simultaneously with the luciferase sensor for the respective miRNA. 24 h after transfection luciferase levels were measured. Data are shown as mean ± S.E., *n* = 4 independent experiments, **, *p* < 0.01. *C, in vivo* inhibition of miRNA-206 induces skeletal muscle hypertrophy. Adult TA muscles were transfected with miRZIP-206. 10 days after electroporation muscles were collected and cryosectioned. Cross-sectional area of transfected fibers, identified by the presence of GFP, was measured. Data are shown as mean ± S.E.; *n* > 700 for each muscle, at least 3 muscles per condition were analyzed; **, *p* < 0.01. *D, in vivo* inhibition of miRNA-21 does not affect skeletal muscle fiber size. Adult TA muscles were transfected with miRZIP-21. 10 days after electroporation muscles were collected and cryosectioned. Muscles were analyzed as described in *C. E,* inhibition of miRNA-206 in denervated muscles partially protects from atrophy. Adult TA muscles were transfected miRZIP-206 and simultaneously denervated and collected 10 days after electroporation. Cross-sectional area were quantified as described in *C*. **, *p* < 0.01. *F,* inhibition of miRNA-21 in denervated muscles partially protects from atrophy. Adult TA muscles were transfected with miRZIP-21 and simultaneously denervated. Muscles were collected after 10 days and the cross-sectional area was quantified as described in *C*. **, *p* < 0.01.

Because these miRNAs are sufficient to induce/exacerbate muscle atrophy, we asked whether they are necessary for the atrophy program and therefore, whether their inhibition would prevent or reduce muscle loss during denervation. We used a vector (miRZIP) that expresses antisense RNAs that act as a sponge for the miRNA of interest. These antisense RNA molecules bind and sequester the miRNAs preventing interaction with the target transcripts. These vectors also co-express the GFP allowing the detection of the transfected fibers. To validate the function of these vectors we performed a luciferase assay. The luciferase sensor of miRNA-206 or miRNA-21 was co-transfected with an empty miRZIP (Zip Null) vector or with the specific miRZIP-206 or miRZIP-21 plasmids. The different miRZIP successfully increased the luciferase activity (Fig. 4, *A* and *B*) of the different sensors confirming their ability to block the target miRNA.

Next we monitored whether inhibition of miRNA-206 and miRNA-21 could reduce atrophy in denervated muscles and promote muscle growth of normal muscles. Thus, we transfected the different miRZIP vectors in innervated and denervated TA. The CSA was measured after 7 days from transfection. The inhibition of miRNA-206 was sufficient to induce 10% hypertrophy of innervated muscle compared with the scramble oligos (Fig. 4*C*). Consistent with the data of miRNA over-expression, inhibition of miRNA-21 under basal conditions did not show any effect on muscle fiber size (Fig. 4*D*). However, inhibition of these miRNAs in denervated muscles was sufficient to partially prevent muscle loss (Fig. 4, *E* and *F*).

##### Identification of miRNA-206 and miRNA-21 Target Genes

**FIGURE 5.**
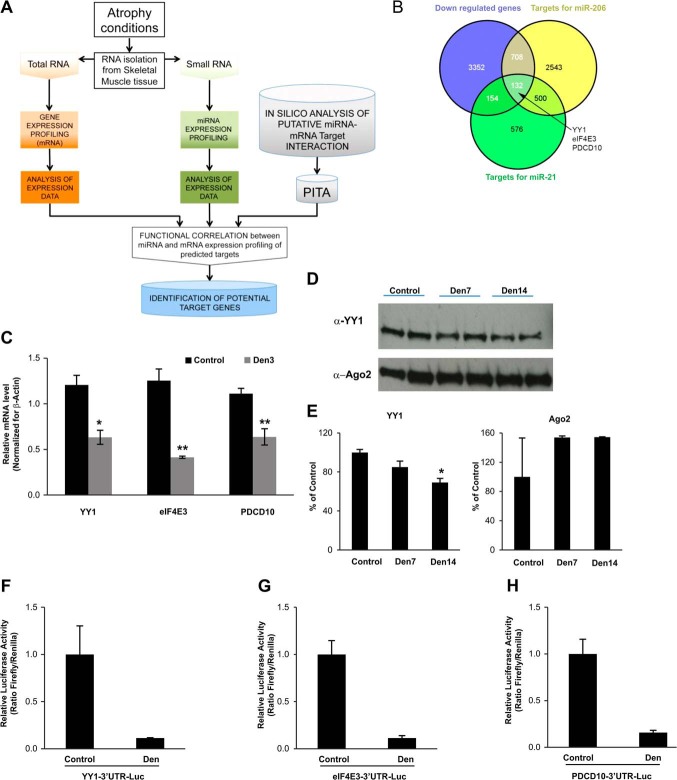
**Approach used to identify biologically relevant targets of miRNA-206 and miRNA-21.** *A,* procedure of computational prediction of miRNA targets. miRNA and gene expression profiles were defined in atrophic muscles of the same mice. Target prediction was performed by the PITA algorithm, then expression data were integrated to improve the detection of functional correlation between miRNA and mRNA expression profiling. *B*, Venn diagram shows the number of down-regulated genes (*blue circle*) in the denervated condition (after 7 and 14 days) that are targets of miR-21 (*green circle*) and miR-206 (*yellow circle*). *C,* denervation down-regulates the expression levels of YY1, eIF4E3, and Pdcd10. qRT-PCR confirmed the down-regulation of YY1, eIF4E3, and Pdcd transcripts after denervation. Data are shown as mean ± S.E. *n* = 3 muscles; *, *p* < 0.05; **, *p* < 0.01. *D*, Western blot for YY1 and Ago2 in TA muscles of control and denervated condition at 7 and 14 days, and *E,* densitometric quantification. The YY1-V5 construct was transfected in C2C12 cells and the lysate was used as positive control for the Western blot. *F–H*, adult tibialis anterior muscles were electroporated with DNA vectors encoding for the luciferase sensor of YY1–3′UTR, eIF4E3–3′UTR, or PDCD10–3′UTR together with *Renilla* luciferase vector to normalize for electroporation efficiency. Simultaneously, the left hindlimb was denervated. 15 days after electroporation/denervation, muscles were collected and luciferase/*Renilla* ratio was determined. Data are shown as mean ± S.E. *n* > 3 independent experiments; *, *p* < 0.05; **, *p* < 0.01.

One of the mechanisms by which miRNAs modulate gene expression is related to mRNA degradation. Several studies demonstrated that depleting cells from specific miRNAs or silencing essential components of the miRNA pathway led to an increase in the abundance of their predicted mRNA targets (39–41). Considering that the algorithms used to predict miRNA targets show a high percentage of false positives, we decided to complement this bioinformatic analysis with biologically relevant data. For this purpose we characterized the gene expression profile of the same denervated muscles that were used to define the miRNA signature. The comparison of the down-regulated genes with the *in silico* predicted targets of miRNA-206 and miRNA-21 significantly reduced the list of the potential targets (Fig. 5*A*). In fact, this analysis defined 840 and 286 potential targets of miRNA-206 and miRNA-21, respectively. Among these genes, only 132 were shared by the two miRNAs (Fig. 5*B* and Tables 5). The first enriched category of both miRNA targets is composed by transcription factors with Yin Yang 1 (YY1) at the top score of the gene list (Table 6). Protein synthesis is another process that is greatly affected by denervation and the eukaryotic translate on initiation factor 4E (eIF4E3) was present in the list (Table 5). Finally, program cell death (PCD) has been described in denervated muscles and program cell death 10 (PDCD10) was a putative target of both microRNAs.

**TABLE 5:** **Anti-correlated genes common to both miRNA-206 and miRNA-21**

Symbol ID	mmu-miR-206		mmu-miR-21	
	Sites	Score	Sites	Score
Acot12	3	−5.49	1	−1.46
Acpp	3	−2.95	1	−1.13
Adam7	1	−9.72	1	−2.8
Adarb2	2	−1.83	2	−4.22
Adipor1	2	−3.94	1	−1.18
Aff4	5	−2.55	1	−1.77
Alg2	1	−6.22	2	−0.52
Ap3m1	3	−0.46	2	−1.93
Atp11a	2	−0.46	1	−1.01
Atp7a	2	−6.75	3	−2.33
Car8	2	−4.38	3	−2.71
Cav1	2	−0.36	2	−1.07
Cav2	4	−2.97	2	−9.2
Ccdc50	3	−7.13	2	−3.51
Ccl9	2	−9.12	1	−4.47
Ccng1	1	−0.4	2	−1.28
Cd2ap	4	−7.98	3	−1.25
Chst11	3	−2.96	1	−0.59
Cited2	2	−7.01	1	−1.78
Clec5a	3	−9.81	3	−4.61
Cmah	1	−2.45	5	−0.68
Col4a1	2	−1.46	1	−1.23
Commd8	2	−8.52	2	−4.71
Copg2	1	−3.29	1	−2.23
Cpd	2	−8.7	4	−1.54
Ddx19b	4	−10.62	3	−6.29
Dkk2	4	−9.29	5	−1.35
Dlg3	4	−2.11	1	−2.21
Dnajb4	1	−8.33	2	−10.58
Dph2	1	−9.24	1	−0.99
Dusp11	3	−11.48	2	−1.42
Ecm2	1	−1.31	2	−1.52
Ehf	1	−2.23	2	−0.096
Eif4e3	1	−3.01	1	−10.9
Esrrg	2	−15.54	3	−5.01
Fgl2	3	−9.23	3	−2.32
Fnbp1	1	−1.68	1	−2.19
Foxo1	3	−4.72	1	−0.28
Fzd7	1	−2.76	3	−0.59
Gabpa	3	−2.58	1	−3.99
Gda	3	−5.35	2	−0.75
Gja1	2	−3.96	1	−1.9
Glis2	2	−8.7	2	−7.31
Gnpnat1	2	−9.86	1	−0.16
Grk1	1	−6.32	8	−4.61
Grpel2	2	−2.88	2	−1.43
Gulp1	1	−3.93	2	−0.1
Hnrnpu	1	−0.86	1	−3.11
Hnrpll	1	−1.17	1	−3.13
Homer1	2	−13.28	2	−0.39
Hs2st1	3	−1.7	2	−0.16
Hs3st3b1	3	−6.53	2	−1.4
Il21	1	−0.19	4	−7.34
Impact	3	−2.14	2	−2.74
Itsn1	7	−3.63	3	−1.09
Map3k7ip3	3	−0.37	3	−3.07
Mbd2	2	−3.14	1	−1.08
Mbnl1	1	−5.8	2	−3.16
Mcfd2	1	−4.5	2	−1.08
Mdfic	3	−4.13	2	−3.8
Mtfr1	1	−3.15	3	−1.49
Myo10	2	−6.75	2	−5.72
Myo5a	3	−4.03	3	−2.32
Ncl	3	−4.45	2	−2.89
Nfib	4	−4.19	5	−6.72
Nras	4	−1.12	3	−0.85
Nrk	2	−0.61	1	−0.76
Ntrk2	5	−5.22	1	−1.54
Oasl2	2	−7.24	1	−4.65
Orc41	3	−1.64	2	−4.97
Pard3	2	−4.04	1	−3.91
Pbx1	1	−9.33	2	−2.96
Pcbp1	1	−3.41	1	−4.93
Pcdh10	2	−2.35	2	−2.12
Pclo	1	−2	2	−1.23
Pdcd10	2	−3.79	1	−2.23
Pdzd2	6	−5.14	3	−2.29
Peli2	5	−8.43	3	−0.57
Pfn2	2	−1.92	2	−0.75
Pitpna	4	−6.9	3	−3.7
Pja2	1	−1.79	2	−1
Plagl1	1	−7.8	3	−0.36
Pls3	2	−7.03	1	−1.84
Ptgs1	1	−2.56	1	−5.16
Pwp1	2	−4.89	1	−1.26
Rab6	1	−1.39	3	−2.68
Rabgap1l	6	−10.08	5	−6.03
Rad51l3	1	−3.95	2	−3.24
Rag2	1	−3.04	2	−0.18
Rbm25	2	−1.25	2	−0.48
Rbpms	3	−0.48	1	−1.3
Rps6kb1	3	−2.9	1	−0.048
Sfrs3	1	−0.65	3	−3.84
Slc1a4	2	−3.31	2	−5.47
Slc25a23	4	−10.07	1	−2.74
Slc25a36	3	−3.11	3	−3.9
Slc35a5	2	−4.24	4	−7.15
Slc4a4	1	−1.73	2	−2.07
Slc7a11	8	−8.43	5	−8.22
Socs5	1	−0.53	2	−2.79
Sox6	7	−8.18	6	−7.92
Srpk2	5	−6.22	3	−0.84
St6gal1	3	−1.78	1	−2.66
Stard5	2	−1.52	2	−1.25
Steap2	7	−9.43	4	−1.91
Stx17	4	−4.52	1	−0.93
Suz12	1	−0.34	2	−4.74
Tbc1d15	1	−9.61	3	−0.75
Tcam1	2	−6.13	1	−1.61
Tm9sf3	2	−1.95	3	−0.98
Tmem100	2	−5.17	2	−0.63
Tmem167	4	−0.95	2	−2.35
Tmod2	8	−8.69	1	−0.28
Trp63	3	−2.25	3	−0.71
Tsc22d2	6	−9.1	3	−4.67
Ubc	1	−14.07	1	−3.42
Ube2g1	2	−12.82	2	−1.86
Usp33	1	−16.97	3	−2.38
Vps37a	9	−7.57	2	−4.96
Wbp2	1	−3.74	1	−6.61
Wtap	1	−4.21	1	−7.76
Yap1	1	−5.83	3	−0.47
Yy1	1	−5.33	2	−8.2
Zbtb33	1	−1.53	4	−2.71
Zcchc8	2	−7.1	2	−1.73
Zfp111	5	−11.5	2	−3.81
Zfp238	1	−4.86	2	−4.54
Zfp36l1	2	−4.72	2	−2.57
Zfp654	4	−5.14	2	−2.76
Zfp704	8	−2.99	6	−3.59
Zfp93	1	−3.34	1	−1.22
Zrsr2	1	−3.49	3	−1.32

**TABLE 6:** **Biological processes found enriched in this gene list**

	Transcription regulation	Zinc finger	Skeletal muscle tissue development	Protein kinase cascade
*p* value	0.0053	0.0098	0.0182	0.0361
Genes	AFF4	ESRRG	CAV1	SRPK2
	CITED2	GLIS2	CAV2	CAV1
	EHF	MBNL1	HOMER1	MDFIC
	ESRRG	PCLO	MBNL1	RPS6KB1
	FOXO1	PJA2		NRK
	GABPA	SUZ12		SOCS5
	GLIS2	UBC		
	MBD2	USP33		
	MDFIC	YY1		
	NCL	ZCCHC8		
	NFIB	ZFP238		
	PBX1	ZFP36L1		
	RBPMS	ZFP654		
	SOX6	ZFP704		
	SUZ12	ZFP93		
	TRP63	ZRSR2		
	YY1	ZBTB33		
	ZFP238			
	ZFP654			
	ZFP93			
	ZBTB33			

**FIGURE 6.**
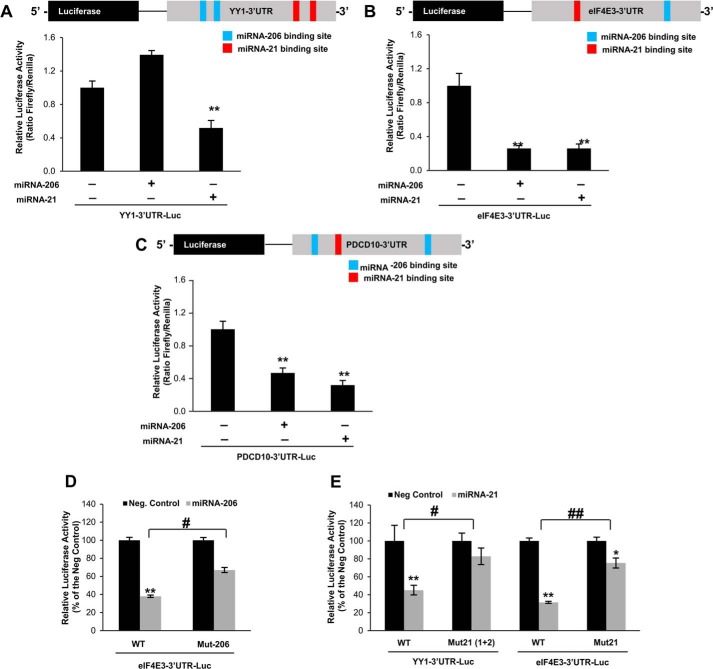
***In vivo* luciferase assays confirm the miRNA-mediated regulation of YY1, eIF4E3, and Pdcd10.** *A,* YY1 is a target of miRNA-21. Adult TA muscles were co-transfected with expression plasmid for miRNA-206 or miRNA-21 and the construct encoding for the luciferase sensor of YY1–3′UTR. A *Renilla* luciferase vector was co-transfected to normalize for electroporation efficiency. Muscles were collected 7 days after electroporation and the luciferase/*Renilla* ratio determined. Data are shown as mean ± S.E., *n* > 3 muscles per condition; **, *p* < 0.01. *B,* eIF4E3 is a target of both miRNA-206 and miRNA-21. Adult TA muscles were transfected and processed as described in *A*; **, *p* < 0.01. *C,* Pdcd10 is a target of both miRNA-206 and miRNA-21. Adult TA muscles were transfected and processed as described in *A*; **, *p* < 0.01. *D,* mutation of the miRNA-206 binding site on the 3′UTR of eIF4E3 partially prevents miRNA-dependent inhibition. C2C12 myoblasts were co-transfected with control or mutated eIF4E3–3′UTR together with scramble or miRNA-206 expressing vector. Luciferase/*Renilla* ratio was determined 24 h after transfection. Data are shown as mean ± S.E., *n* = 3 independent experiments; **, *p* < 0.01; #, *p* < 0.05. *E*, mutation of the miRNA-21 binding sites on the 3′UTR of YY1 and eIF4E3 partially prevent miRNA-dependent inhibition. C2C12 myoblasts were co-transfected with the control or mutated version of the 3′UTR of YY1 or eIF4E3 together with scramble or miRNA-21 expressing vector. Luciferase/*Renilla* ratio was determined 24 h after transfection. Data are shown as mean ± S.E., *n* = 3 independent experiments; *, *p* < 0.05; **, *p* < 0.01; #, *p* < 0.05; ##, *p* < 0.01.

**FIGURE 7.**
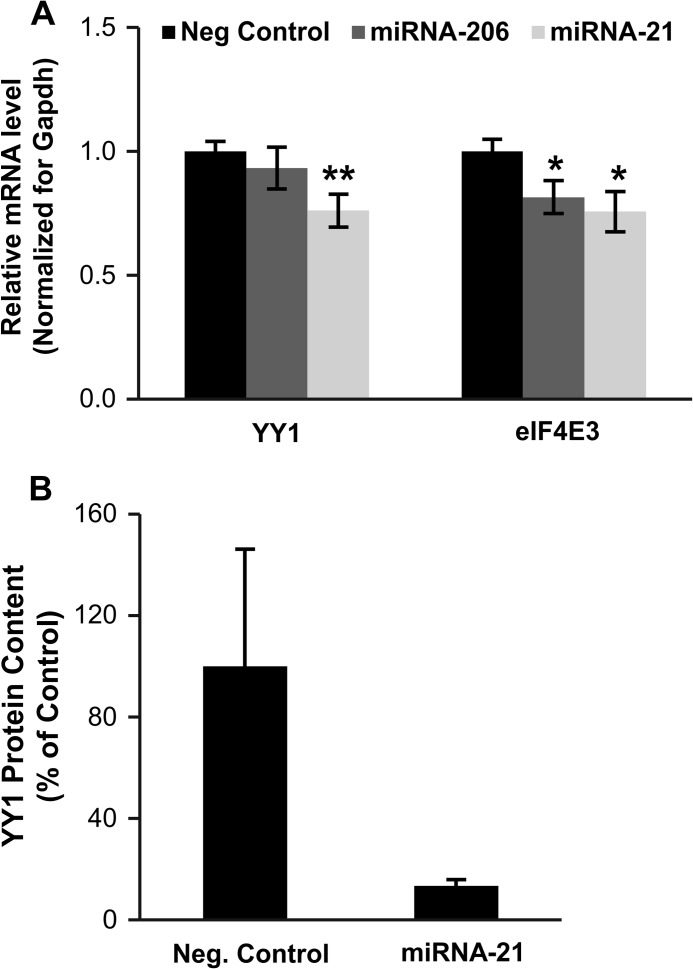
*A*, over-expression of miRNA-206 or miRNA-21 directly regulates the mRNA of YY1 and eIF4E3. C2C12 myoblasts were transfected with miRNA-206 or miRNA-21 expressing vector. RNA was extracted 48 h after transfection and the expression levels of YY1 and eIF4E3 were analyzed by qRT-PCR. Data are shown as mean ± S.E., *n* = 3 independent experiments; *, *p* < 0.05; **, *p* < 0.01. *B,* densitometric quantification of the Western blot for YY1 in C2C12 myoblasts over-expressing negative control or miRNA-21. Data are shown as mean ± S.E., *n* = 3 independent experiments.

Quantitative RT-PCR confirmed that YY1, eIF4E3, and PDCD10 transcripts were down-regulated in denervated muscles (Fig. 5*C*). Down-regulation of the YY1 protein in denervated muscles was confirmed by Western blot analyses. Interestingly, AGO2 expression was not affected by this catabolic state (Fig. 5, *D* and *E*). We could not find commercial antibodies that were specific for PDCD10 and eIF4E3. To confirm that these genes were targets of these miRNAs we performed luciferase reporter assay. The 3′UTR of the genes of interest was cloned into the pMIR vector downstream the luciferase gene. Next these 3′UTR-luciferase sensors were transfected into control and denervated TA muscles. A significant reduction of the luciferase activity was detected in the denervated muscles suggesting that the 3′UTR of these genes are under miRNA control (Fig. 5, *F–H*). To demonstrate that miRNA-206 and miRNA-21 were involved in this regulation, we transfected adult TA muscle with the 3′UTR-luciferase sensors together with the miRNA expressing vectors. Interestingly, 7 days later the expression of miRNA-206 reduced the 3′UTR-luciferase activity of eIF4E3 and PCDC10 but not of YY1 (Fig. 6, *A–C*). The expression of miRNA-21 reduced the activity of all the 3′UTR-luciferase sensors (Fig. 6, *A–C*). Altogether, these *in vivo* results demonstrate that YY1 is modulated by miRNA-21, whereas eIF4E3 and Pdcd10 are modulated by both miRNA-206 and miRNA-21. To further prove the microRNAs binding, we mutagenized the 3′UTR binding sites of YY1 and eIF4E3. The mutated sensors were transfected into adult muscles together with the expression vectors for miRNA-206 and miRNA-21. The activities of the mutated 3′UTR-luciferase sensors were totally or partially restored when expressed together with the microRNAs (Fig. 6, *D* and *E*). These results confirm that the 3′UTR of YY1 is regulated by miRNA-21, whereas the 3′UTR of eIF4E3 is regulated by both miRNA-206 and miRNA-21. Consistently, transfection of C2C12 muscle cells with miRNA-206 led to a significant down-regulation of eIF4E3 transcript (Fig. 7*A*), whereas over-expression of miRNA-21 led to a significant decrease of YY1 and eIF4E3 mRNAs (Fig. 7*A*). Importantly, over-expression of miRNA-21 led to down-regulation of the YY1 protein (Fig. 7*B*). In conclusion, miRNA-206 and miRNA-21 are important for fine-tuning the denervation atrophy program and their modulation could be a novel potential therapeutic approach to counteract muscle loss and weakness in catabolic conditions.

## DISCUSSION

Our data demonstrate that miRNA expression is affected by catabolic conditions and modulates the atrophy program after denervation. Moreover, miRNA induction in denervated muscles is delayed when compared with the transcriptional regulation. In fact 3 days after denervation is a time in which muscles are not yet atrophic and are characterized by the peak of mRNAs induction while miRNAs are mainly suppressed. Instead at 7 days of denervation muscles are already atrophic and miRNAs are induced. Altogether these findings suggest that microRNAs are involved in the fine-tuning of an already initiated atrophy program. This concept is also sustained by the minor changes that miRNA-206 and miRNA-21 over-expression and inhibition elicited on myofiber size. A similar observation was achieved in human muscle stem cell (42). Accordingly, miRNAs were globally down-regulated in quiescent compared with proliferating satellite cells. The recent findings that DICER1 and EIF2C/AGO, two essential components of the miRNA machinery, are substrates of the autophagy-lysosome system define a link between autophagy activation and suppression of microRNA maturation (43). Because autophagy is activated at early stages of muscle atrophy (44–47) it is possible that suppression of miRNA biogenesis and processing depends on autophagy-dependent degradation of DICER and AGO. However, when we looked at AGO2 expression we could not find a significant down-regulation at 7 days of denervation.

Two other studies conducted in different models of cardiac hypertrophy (48) and in several inherited muscular disorders (22) have analyzed the miRNA signature and identified a small subset of miRNAs commonly induced during these diseases. However, it is worth noting that most of the deregulated miRNAs in cardiac hypertrophy or in inherited muscle diseases were specific of each pathological condition. This is in line with our data that did not identify a common signature of miRNA in different models of muscle atrophy. The peculiarity of the miRNA signature for each atrophic condition further confirms the concept of a specific fine-tuning/regulation of an activated atrophy process.

This is the first study that compared miRNAs expression in early stages of muscle atrophy and dissected their function, *in vivo*. Other studies described the changes that occur in diabetes (49), long term denervation/reinnervation (50, 51), and dexamethasone-induced atrophy(52). The decision to focus on denervation was driven by the fact that this model displayed the strongest induction of microRNAs. Moreover, despite the fact that transcriptomes of denervated muscles are well characterized (6, 35, 53), miRNA expression is still unexplored. A few published studies have defined the microRNA profile in long term denervated muscles (1 and 4 months)(50, 51). However, these muscles are in fully established atrophy and therefore, the changes of miRNA profile are mainly related to metabolic adaptations/compensations that follow such long inactivity. Instead, our analyses at early time points unraveled the role of miRNA before and/or during the early stages of atrophy program. This approach allows us to identify miRNA-206 and miRNA-21 as important modulators of muscle loss.

miRNA-206 is a muscle-specific microRNA that has been found to be involved in myoblast differentiation (36, 54), muscle regeneration (21), and nerve growth after injury (24). Previous work identified miRNA206 as a critical factor of muscle reinnervation in SOD1^G93A^ transgenic mice, an animal model of amiotrophic lateral sclerosis that is characterized by motor-neuron degeneration. Despite the evident effect of miRNA-206 deletion in SOD1^G93A^ transgenic animals, miRNA-206 knock-out mice did not show any evident phenotype in basal conditions (24). However, a compensation operated by miRNA-1, another muscle-specific miRNA, may account for the lack of phenotype in these knock-out mice. In fact miRNA-206 and miRNA-1 share the same seed region and differ only in 3 nucleotides at the 3′-end region (55), suggesting that they possibly regulate the same set of mRNAs targets and therefore, possibly identical biological processes. By using an electroporation technique, we acutely perturbed the miRNA content of adult muscle minimizing compensatory mechanisms. Our findings are consistent with a recent study that identified miRNA-206 as an important player in myotube size. However, such changes were not confirmed when the authors moved to *in vivo* experiments (56). The discrepancy of this result with our data could be explained with the level of expression of miRNA-206. In this study the authors used AAV-mediated delivery of miRNA-206 that resulted in a 20- and 100-fold increase of miRNA206 in muscles. Our transfection approach gave a 2–3-fold up-regulation of mature miRNAs mimicking the endogenous induction that occurs in denervated muscles. Therefore, our experimental condition did not saturate the miRNA machinery reducing the possibility of off-target effects.

MicroRNA-21 is mainly known for its anti-apoptotic and oncogenic potential (57). miRNA-21 is also important in the cardiovascular system because it is highly up-regulated during cardiac stress, but its actual role is still controversial. Interestingly miRNA-21 was induced in 8 of 10 inherited muscular diseases (22) suggesting that it can be part of the complex mechanisms of muscle wasting. However, when we overexpressed miRNA-21 in innervated muscle we did not induce muscle atrophy suggesting that miRNA-21 requires the co-expression of other microRNAs to fully elicit an atrophic action. The kinetic of miRNA-21 and -206 induction in denervated muscles suggests that miRNA-206 is one of the miRNA-21 partners and that the simultaneous induction of both microRNAs is critical for an optimal down-regulation of important targets controlling muscle mass. In fact miRNA-21 is already up-regulated at 3 days of denervation, a time in which muscles are not atrophic and miRNA-206 is not significantly induced. Importantly, at 7 days both miRNAs are induced and muscles are 25% smaller than controls. This was confirmed by experiments in which miRNA-206 and miRNA-21 were co-expressed leading to an exacerbation of muscle atrophy induced by just miRNA-206 expression.^6^

To understand which mechanisms are controlled by these miRNAs we performed an mRNAs expression profile in parallel to the miRNAs expression profile. Among the statistically significant activated pathways in denervated muscles we identified the proteasome, phagosome, protein digestion, and absorption pathways (Table 7), whereas metabolic pathways like the insulin signaling pathway was turned off (Table 8) (58, 59). The microarray analyses identified 4,346 transcripts down-regulated between 3 days of denervation and 7 or 14 days (Fig. 5*B*), an opposite profile of that of miRNA-21 and -206. The comparison of down-regulated transcripts with the predicted miRNA targets led to a list of candidates that were further reduced when we selected transcripts regulated by both miRNAs. This strategy led to the identification of YY1, eIF4E3, and PDCD10. Although the function of these genes in atrophy is unclear or unknown, we confirmed their miRNA-dependent suppression in denervation. The YY1 transcription factor is involved in mitochondrial biogenesis in adult muscles (60) through the interaction with PGC1a. Inactivation of YY1 in muscle causes abnormalities of mitochondrial morphology and oxidative function associated with exercise intolerance (61, 62). This finding opens the possibility that miRNA-21, through the regulation of YY1, participates in the regulation of mitochondria biogenesis/functioning. In fact, it is well established that loss of innervation causes a suppression of mitochondrial function and β-oxidative metabolism (6, 63, 64). Therefore, microRNA-dependent suppression of YY1 can contribute to these metabolic adaptations after denervation.

**TABLE 7:** **Pathways activated during denervation**

Activated	Proteosome	Phagosome	MAPK signaling pathway	Ribosome
*p* value	1.52E-13	1.03E-09	6.47E-06	8.75E-06
Genes	Psmb4	Atp6v0e	Flnc	Rps18
	Psmc1	Itgb1	Cacng1	Rps5
	Psmb1	Thbs3	Traf2	Rpl22
	Psma7	Atp6v1g1	Relb	Rpl23
	Psmb5	H2-Eb1	Mknk2	Rps10
	Psmd4	Atp6v1e1	Gadd45a	Rps9
	Psmd2	Tubb6	Rras2	Rpl10a
	Psmd14	Calr	Hspb1	
	Psma4	Tap1	Rras	
	Psmc4	Rab5a	Mapt	
		Tubb2a		
		Tuba1b		

**TABLE 8:** **Pathways inhibited during denervation**

Inhibited	Metabolic pathways		PPAR signaling pathway	Amyotrophic lateral sclerosis (ALS)	Insulin signaling pathway
*p* value	5.34E-08	7.53E-08	2.20E-06	4.65E-06	
Genes	Abo	Got1	Acadm	Casp12	Mapk1
	Acadm	Hmgcr	Cd36	Grin1	Phka1
	Adk	Ldhb	Cpt1b	Grin2b	Ppargc1a
	Aldh6a1	Mat2b	Cyp27a1	Nos1	Ppp1cb
	Amd1	Mccc1	Fabp3	Ppp3r1	Ppp1r3c
	Bpgm	Mpst	Lpl	Slc1a2	Slc2a4
	Cs	Ndst3	Ppara		Socs1
	Csl	Nnt	Rxra		Srebf1
	Cyp27a1	Nos1			
	Cyp2c65	Pla2g7			
	Dbt	Prodh			
	Dhcr24	Prodh2			
	Ephx2	St3gal6			
	Galt				

The initiator factor eIF4e3 is one of the most critical factors that regulate ribosome assembly and protein synthesis. Importantly, eukaryotic translation initiation factor 4E-binding protein 1 (eIF4e3-BP1 or simply 4EBP1) is a repressor of protein synthesis and belongs to the group of atrophy-related genes(59). The transcriptional-dependent up-regulation of 4EBP1 and the miRNA-dependent inhibition of eIF4E3 suggest a coordinated action to finely tune protein synthesis in denervated muscles. Moreover, our finding that two miRNAs control eIF4E3 expression is consistent with the concept that miRNA-206 and miRNA-21 synergistically act to slow down the rate of protein synthesis. We cannot exclude that other targets are involved in the regulation of muscle mass. Especially the data that miRNA-206 alone is sufficient to induce atrophy may support this concept. Indeed, the potential miRNA-206 targets include factors, such as Smad1, Runx1, JunD, and Rheb, that have been described to affect muscle mass. The contribution of miRNA-206 in the regulation of these targets will be evaluated in the future. In conclusion, our data propose the novel hypothesis that miRNAs act as important players in the modulation of the atrophy program that controls critical components of mitochondria function and protein synthesis.

## Footnotes

6 R. J. Soares, S. Cagnin, F. Chemello, M. Silvestrin, A. Musaro, C. De Pitta, G. Lanfranchi, and M. Sandri, unpublished data.

## Footnotes

5 The abbreviations used are:

miRNA: micro RNA
TA: tibialis anterior
qRT: quantitative RT
PCD: program cell death.
